# Change of Oxidation Mechanisms by Laser Chemical Machined Rim Zone Modifications of 42CrMo4 Steel

**DOI:** 10.3390/ma14143910

**Published:** 2021-07-13

**Authors:** Alexander Schupp, René Daniel Pütz, Oliver Beyss, Lucas-Hermann Beste, Tim Radel, Daniela Zander

**Affiliations:** 1 Chair of Corrosion and Corrosion Protection, Foundry Institute, RWTH Aachen University, Intzestr. 5, 52072 Aachen, Germany; a.schupp@gi.rwth-aachen.de (A.S.); r.puetz@gi.rwth-aachen.de (R.D.P.); o.beyss@gi.rwth-aachen.de (O.B.); 2 BIAS-Bremer Institut für Angewandte Strahltechnik GmbH, Klagenfurter Str. 5, 28359 Bremen, Germany; beste@bias.de (L.-H.B.); radel@bias.de (T.R.)

**Keywords:** LCM, 42CrMo4, in situ XRD, oxidation, rim zone, XPS, oxidation mechanism

## Abstract

The oxidation mechanism of metals depends, among other factors, on the surface integrity. The surface and rim zone properties are often determined by the manufacturing process that was used to machine the material. Laser chemical machining (LCM) is a manufacturing process that uses laser radiation as a localized and selective heat source to activate a chemical reaction between an electrolyte and a metallic surface. The objective of this work is first to investigate how different LCM processes affect the rim zone properties of 42CrMo4. For this purpose, the surface chemistry is analyzed by EDS and XPS, phases and residual stresses are determined by XRD, and the morphology is investigated by SEM. Second, the influence of these modified rim zones on the oxidation properties of the steel at 500 °C in air is to be demonstrated in oxidation tests by in situ XRD and subsequent SEM/EDS investigations. A decisive influence of the oxides formed on the surface of 42CrMo4 during LCM in different electrolytes (NaNO_3_ solution and H_3_PO_4_) at two different laser powers on the high-temperature oxidation properties was demonstrated. These oxides were supposed to act as nucleation sites for oxide layer formation at 500 °C and led to an overall increase in oxide layer thickness after high-temperature oxidation compared to non-LCM-processed surfaces.

## 1. Introduction

The use of materials for engine components, such as cylinders, requires both excellent mechanical properties and sufficient high-temperature corrosion resistance. It has been reported that the high-temperature oxidation behavior of 42CrMo4 steel, which is used for crankshafts, for example, depends on rim zone properties such as residual stresses, roughness and chemical composition [1]. These rim zone properties are determined by the mechanical, thermal, thermo-chemical or chemical loads of the applied manufacturing process. The laser chemical machining (LCM) of metals is used for manufacturing difficult to machine materials and surface finishing [2,3]. Thermo-chemical loads which occur during LCM typically result in a change of roughness, residual stresses and surface chemical composition [4,5]. All are expected to have a significant influence on oxidation mechanisms and thus also on oxidation resistance. However, the relationships between LCM-manufactured rim zone properties and oxidation mechanisms of 42CrMo4 steel at high temperatures are not fully understood yet.

LCM is an unconventional ablation process [6], which combines the techniques of laser processing and chemical processing and is mostly used for passivating materials, such as titanium-based alloys. The process is operated by laser radiation as a localized and selective heat source to activate a heterogeneous chemical reaction between a liquid medium and a metallic surface via a suitable thermal effect. The local heat-activation results in a temperature-induced electrochemical metal dissolution [7,8] and a temporary breakdown of passive compounds and layers at the metal surface [9]. Since the laser beam enables selective, precise and contact-free processing, laser chemical processing has a high degree of flexibility and is used in a wide range of processing concepts. One of these is the scanner-based laser chemical polishing. In this process, the laser beam is moved over the workpiece surface at high velocities by a scanner. The scan velocities can vary between 1 and 20 mm/s [3]. Electrolyte boiling [10] and the resulting bubbles are recognized as disturbing influencing variables for laser chemical processing. Since temperatures above the boiling temperature are quickly reached even at low laser intensities, a boiling process is generated. However, this can be reduced by pressure, for example [11].

Investigations on the processing of 42CrMo4 steel by LCM demonstrated that the selection of suitable process parameters (laser power, process electrolyte) is challenging [5,12]. Thus, when comparing the electrolyte NaNO_3_ solution, H_3_PO_4_ and water, sufficiently high ablation rates could only be achieved in H_3_PO_4_, but at the expense of geometric accuracy. This is attributed to the lack of the formation of a stable passive layer formed by 42CrMo4 steel which makes it difficult to remove material with the same precision as is possible with passivating titanium, for example [5,13]. In NaNO_3_ solution, only low material removal rates can be achieved at low laser powers (below 8–9 W [5]). At higher laser powers, the material removal rate can be increased. However, this occurs at the expense of partial melting of the surface [5,12]. This is also in accordance with a fundamental study of the influence of LCM on stainless steel in NaNO_3_ solution [14]. Furthermore, Eckert et al. [5] demonstrated a change of the rim zone properties introduced by LCM and dependent on the process parameters. However, the influence of changed rim zone properties on the surface functionality at high temperatures of, e.g., below the wüstite temperature in air was not investigated.

The oxidation behavior of iron and low-alloyed steels in air at high temperatures has already been investigated for decades and strongly depends on alloy and surface composition as well as microstructure [15,16]. It is generally accepted that during the oxidation of pure iron at temperatures around 500 °C, the oxides hematite (Fe_2_O_3_) and magnetite (Fe_3_O_4_) are thermodynamically stable. A Fe_3_O_4_ layer will be formed at the interface iron/Fe_2_O_3_ layer due to the lower dissociation pressure of Fe_3_O_4_ compared to Fe_2_O_3_ [17,18]. The oxide growth is driven by diffusion and follows a parabolic law. The diffusion of Fe^2+^ and Fe^3+^ through the oxide towards the interface oxide/atmosphere is known as the rate-determining step for the formation of the outer Fe_2_O_3_ layer. Contrastingly, the formation of the inner Fe_3_O_4_ layer is influenced either by the outwards directed metal ion diffusion or the inwards directed oxygen diffusion dependent on the partial oxygen pressure [18,19].

In the case of low-alloyed chromium-molybdenum steels, e.g., 1.25Cr-0.5Mo and 2.25Cr-1Mo, an additional third layer forms during oxidation in air at 500 °C. This layer is formed at the interface Fe_3_O_4_/steel and is composed of magnetite and iron-chromium spinel [20]. This oxide is significantly enriched in chromium, but also in other elements such as silicon, compared to the upper Fe_2_O_3_ and Fe_3_O_4_ layers. This was also demonstrated by Folkeson et al. [21] for the oxidation of 2.25Cr-1Mo in an atmosphere containing 5% oxygen and 40% humidity at 400 and 500 °C, respectively. Furthermore, Trindade et al. [18] determined that the chromium content within the magnetite layer increases towards the metal for 2.25Cr1Mo in laboratory air at 550 °C. Depending on the chromium content and oxygen partial pressure, FeCr_2_O_4_ and Cr_2_O_3_ was detected. It was summarized that an increased chromium content in the oxide shows a positive effect on the oxidation resistance of the steel below the wüstite temperature.

Furthermore, the formation and growth of the oxide layers of steels depend strongly on microstructural and surface parameters close to the rim zone, such as residual stresses, roughness and surface chemistry. Bae et al. [22] were able to demonstrate for welds on P122 Cr-Mo steel that the residual stresses and microstructural changes, introduced as a result of welding, have an effect on oxidation resistance. For example, oxidation at 600 °C in air showed that the oxidation resistance of the heat-affected zone along the welds is lower than that of the base material. The formation of needle-shaped oxides was identified, which occur in regions that exhibit residual stresses.

It is also known that residual stresses influence the diffusion rates of alloying elements close to the rim zone and affect the oxide growth. It was reported by Raceanu et al. [23] that compressive residual stresses exhibit a negative effect on diffusion rates due to the compression of the crystal lattices, whereas tensile stresses lead to an expansion of the crystal lattice and thus to accelerated diffusion. This effect was demonstrated for the oxidation of zirconium, where compressive residual stresses were introduced into the surface by shot-peening.

The influence of rime zone modifications, such as residual stresses and roughness, on the oxidation of 42CrMo4 steel has been studied in oxygen at a temperature of 600 °C by Zander et al. [1]. It was demonstrated that the changes in the rim zone properties introduced by electrochemical machining (ECM), electric discharge machining (EDM) and grinding significantly influence the oxidation mechanisms. A three-layer oxide was detected on all three surfaces after 24 h of oxidation: an inner layer consisting of Fe_3_O_4_ + Fe/Fe_1−x_O, a middle layer of Fe_3_O_4_ and an outer layer of Fe_2_O_3_. An enrichment of the inner layer with chromium and silicon was detected as well. However, after 24 h of oxidation, the ECM surfaces were found to reveal an increased oxide layer thickness and mass compared to the ground and EDM surfaces. This was related to the different residual stresses and roughness states of the different surfaces. An increase in active surface thereby increases oxidation rate, whereas compressive stresses are assumed to have a positive impact on the oxidation resistance at 600 °C.

In addition to residual stresses, surface chemistry is also relevant for the formation of oxides at high temperatures. Brito et al. [24] were able to demonstrate on Fe-Al that Fe_2_O_3_ can serve as nucleation sites for Al_2_O_3_ layer growth. This leads to the preferential formation of dense, well-protective layers, which reveals a positive effect on the oxidation resistance. Numerous other studies for iron and steels focused on the positive effect of applying oxides on the surface as nucleation sites for the formation of stable oxide as barrier layers to increase the oxidation resistance of the metal at high temperatures. This includes, among others, the application of cerium oxides to metallic surfaces, which can serve as nucleation sites for the oxide film formation [25,26,27].

The aim of the present work is to investigate the influence of rim zone properties generated by LCM on the oxidation mechanism of 42CrMo4 steel at 500 °C in air. This is to be used to specifically modify rim zones with regard to their oxidation properties by means of LCM. For this purpose, selected rim zone properties, such as residual stresses and the chemical composition of surfaces machined by LCM in NaNO_3_ solution and in H_3_PO_4_ at two different laser powers, were analyzed. Subsequently, LCM surfaces were oxidized in air and compared to a ground surface after oxidation at 500 °C for 20 h. It is expected that the modification of the rim zone properties by LCM will lead to a change in the oxidation behavior of 42CrMo4 steel.

## 2. Materials and Methods

### 2.1. Materials

42CrMo4 steel (AISI 4140) (Deutsche Edelstahlwerke GmbH, Witten, Germany) was investigated as a low-alloyed heat-treatable steel within this study. The main alloying elements are chromium, manganese, carbon, silicon and molybdenum, as demonstrated in Table 1.

The material exhibits a martensitic microstructure obtained by a heat treatment at 850 °C for 2 h in a vacuum furnace and quenching in oil to 60 °C. Afterwards, the steel was tempered at 400 °C for 4 h and then cooled in air to room temperature. The heat-treated material reveals a yield strength of 1430 MPa, a tensile strength of 1570 MPa and a hardness of 470 HV0.2. The material was cut into small cylinders with a height of 1.9 mm and a diameter of 12 mm by using abrasive waterjet cutting and a cutting wheel. After that, the steel was embedded in a non-conductive embedding material (KEM 35). Then, one face of the cylinders was ground to a grit of 1000 with SiC sandpaper using a contact force of 15 N and a grinding wheel rotation speed of 150 rpm.

### 2.2. LCM

**Figure 1:**
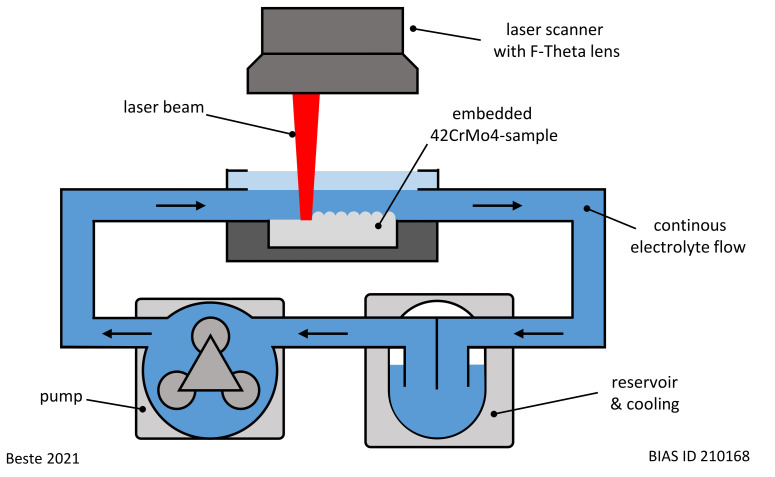
Experimental setup used for LCM.

The embedded samples were processed by LCM. The experimental setup shown in Figure 1 was used for this purpose. A laser (wavelength: 1080 nm, spot diameter: 110 µm) was applied to scan the surface with a laser spot speed of 10 mm/s and a trajectory offset of 20 µm. In total, an area of 10 × 5 mm^2^ on each surface was laser scanned ten times.

The process parameters of LCM are given in Table 2. Two types of electrolytes were used for the processing: sodium nitrate (NaNO_3_) solution (Carl Roth GmbH + Co. KG, Karlsruhe, Germany) and phosphoric acid (H_3_PO_4_) (Carl Roth GmbH + Co. KG, Karlsruhe, Germany). In each case, the electrolyte was circulated for one experiment at room temperature. A ground state was also investigated for comparison. Thus, a total of four different surfaces were analyzed in the course of this work.

### 2.3. Methods

The microstructure and chemical composition of the surfaces were characterized by scanning electron microscopy (SEM) using a Zeiss Supra 55 VP (Carl Zeiss Microscopy Deutschland GmbH, Oberkochen, Germany), equipped with an energy-dispersive X-ray spectroscopy facility (EDS) from Oxford Instruments (Oxford Instruments plc., Abingdon, UK). For cross-sections, backscattered electrons (BSE) were used for imaging. The acceleration voltage was adjusted to 15 kV and a working distance of approximately 10 mm was used. On the cross-sections, additional EDS line scans were performed. For top-view SEM images, secondary electrons (SE) at an acceleration voltage of 5 kV and a working distance of approximately 5 mm were used.

A Kratos Axis Supra (Kratos Analytical Ltd., Manchester, UK) X-ray photoelectron spectroscope (XPS) was used to obtain information on the chemical composition of the studied rim zone and on the bonding states of the elements. Therefore, a monochromatic Al KαX-ray source (hν = 1486.6 eV) was utilized. XPS spectra of the O1s, C1s, Fe2p, Cr2p, Mo3d, N1s and P2p regions were recorded with a pass energy of 20 eV and a step size of 0.1 eV. The evaluation of the XPS spectra with respect to the chemical composition of the investigated edge zones as well as the present bonding conditions was carried out with the software ESCApe (Kratos Analytical Ltd., Manchester, UK).

All peaks were charge corrected by assigning the C-C component in the C1s peak to 284.8 eV. As already presented in previous publications by the authors [29,30], Shirley-type backgrounds and Gauss-Lorentz (30/70)-type line shapes were used for peak fitting for all components except metallic iron (asymmetric line shapes).

Ex situ phase analysis and residual stress measurements were performed before oxidation using a Panalytical Empyrean X-ray diffractometer (XRD) from Malvern Panalytical Ltd. (Malvern, UK) with Co-Kα radiation (40 kV, 40 mA). The Panalytical High Score Plus software (Malvern Panalytical Ltd., Malvern, UK) with ICDD databases was used to determine the different phases. For phase analysis, a 2θ range of 30–75° was measured at predefined time intervals at an incidence angle of 10°. A step size of 0.1° and a measuring time of 2 s per step was used. The reference cards ICDD: 98-018-3975 (Fe_3_O_4_), ICDD: 98-041-5251 (Fe_2_O_3_), ICDD: 98-005-3451 (α-Fe), and ICDD: 98-018-6833 (γ-Fe) were finally selected for phase evaluation. In addition, residual stress measurements were carried out by sin Ψ² method on the α-Fe (112) peak according to Zander et al. [1].

In situ XRD measurements were conducted during high temperature oxidation to determine the evolution of the oxide layers. The surfaces that have been machined by LCM or by grinding were oxidized for 20 h at 500 °C in ambient air in a XRK 900 high temperature oxidation chamber (Anton Paar Germany GmbH, Ostfildern-Scharnhausen, Germany). The heating rate was 50 K/min. Once the target temperature was reached, the height of the sample holder had to be beam adjusted due to the thermal expansion of the material and the sample holder. This took approximately 5 min. Therefore, the first in situ XRD measurement started after 5 min of oxidation at 500 °C. During oxidation, the evolution and growth kinetics of the oxide layer were investigated by in situ XRD. To obtain information on the oxidation kinetics, the summation method was chosen, which has already been described by Czech et al. [31]. The intensity of all peaks of one species (e.g., Fe_2_O_3_, Fe_3_O_4_) in the measuring range is summed up and plotted against time. All counts within the respective peak range are summed up after the background has been subtracted. After 20 h of oxidation, the sample was cooled at a rate of 30 K/min in the high-temperature chamber.

## 3. Results

### 3.1. Surface and Rim Zone Analysis before Oxidation

**Figure 2:**
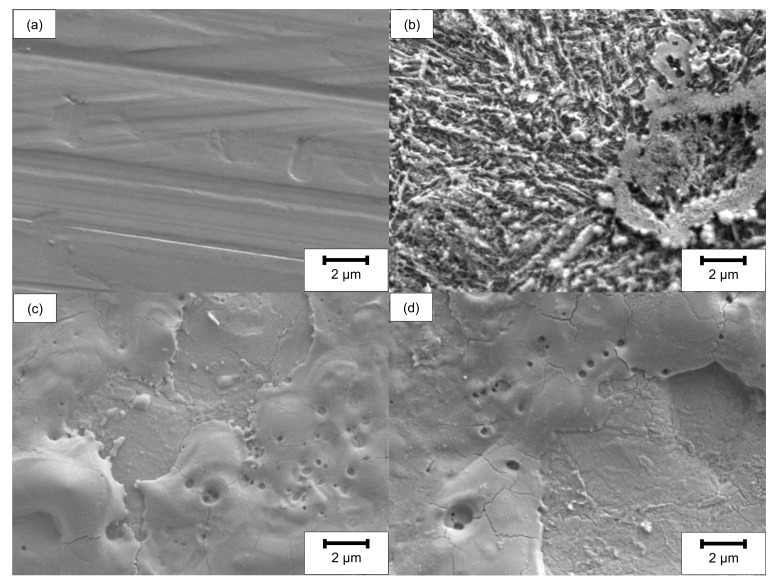
SEM top-view images before oxidation of (**a**) ground, (**b**) LCM-H_3_PO_4_-6W, (**c**) LCM-NaNO_3_-6W and (**d**) LCM-NaNO_3_-18W 42CrMo4 steel.

The surface and rim zone analysis of 42CrMo4 steel for the LCM machined surfaces and the ground surface was performed by SEM and XPS before oxidation. Figure 2 shows SEM top-view images of all surfaces. In contrast to the ground specimen, the surface machined by LCM in H_3_PO_4_ with a laser power of 6 W (LCM-H_3_PO_4_-6W) reveals an etched, martensitic microstructure of the 42CrMo4 steel with some deposits on the surface.

The surfaces machined in NaNO_3_ solution using LCM at 6 W (LCM-NaNO_3_-6W) and 18 W (LCM-NaNO_3_-18W) are considerably different in comparison to the ground and the LCM-H_3_PO_4_-6W surfaces (Figure 2c,d). The surfaces are partially covered with a porous layer. No etching of the martensitic microstructure was observed, but the formation of a layer consisting of pores as well as areas of spalling was observed.

**Figure 3:**
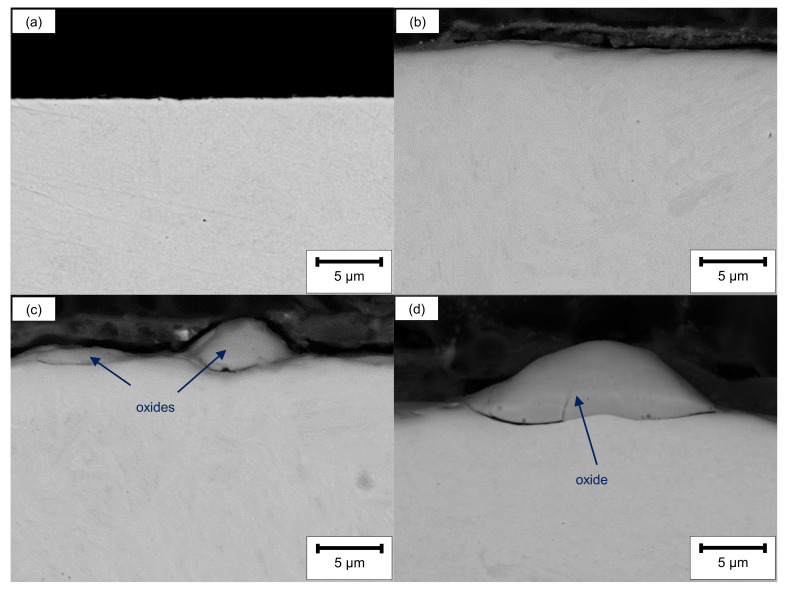
SEM cross-sections before oxidation of (**a**) ground, (**b**) LCM-H_3_PO_4_-6W, (**c**) LCM-NaNO_3_-6W and (**d**) LCM-NaNO_3_-18W 42CrMo4 steel.

SEM cross-sectional analysis revealed no significant differences between the ground and the LCM-H_3_PO_4_-6W surface (Figure 3a,b). This is attributed to the influence of the edge effect on the excitation volume as well as on the resolution of SEM. However, a partially hill-shaped and irregularly covered surface layer was observed on both LCM-NaNO_3_-6W and LCM-NaNO_3_-18W (Figure 3c,d) with a maximum thickness of about 10 µm. We observed almost no influence of the laser power on the appearance of the surface layer for LCM in NaNO_3_.

**Figure 4:**
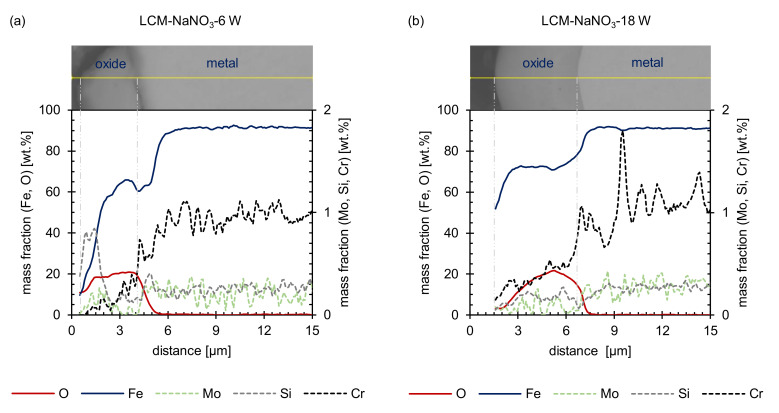
EDS line scans before oxidation: (**a**) LCM-NaNO_3_-6W and (**b**) LCM-NaNO_3_-18W 42CrMo4 steel.

Additional EDS line scans of the LCM-NaNO_3_-6W and LCM-NaNO_3_-18W cross-sections confirmed that the hill shaped layer mainly consists of iron oxide (Figure 4). Considering that a stoichiometrically idealized Fe_3_O_4_ oxide consists of 20 wt.% oxygen and 80 wt.% iron, the EDS analysis reveals the formation of a Fe_3_O_4_ with a minor deficit in iron for both surface states. In addition, chromium, molybdenum and silicon were observed within the oxide layer. Some enrichment of silicon close to the oxide surface is attributed to the polishing procedure and the embedding material.

XPS investigations of the ground and LCM surfaces give more detailed information on the chemical composition for the first nm in depth of the surface layers (Table 3). In contrast to the SEM investigations, it was possible to observe the formation of a layer for all surfaces. The surface of the layers mainly consists of iron and oxygen. All LCM samples have in common that the surfaces are enriched in chromium compared to ground 42CrMo4 steel. Furthermore, a significant molybdenum enrichment as well as phosphorus was observed at the LCM-H_3_PO_4_-6W surface.

**Figure 5:**
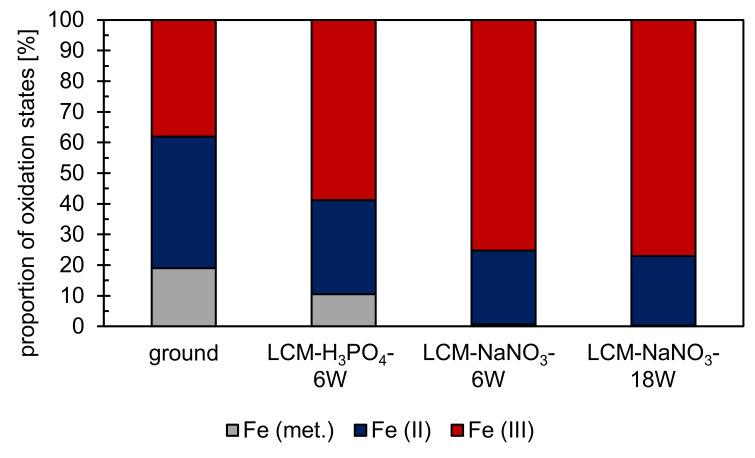
XPS analysis on the proportion of iron oxidation states at the rim zone after surface finishing by LCM and grinding. XPS results of the ground surface were adapted with permission from ref. [29]. Copyright 2021 Zander et al., licensee MDPI, Basel, Switzerland. XPS fitting of iron was performed according to Grosvenor et al. [32] and Biesinger et al. [33].

In addition to the chemical composition, XPS also revealed the stoichiometric composition and structure of the uppermost nm of the layer on the machined surfaces. Therefore, the oxidation states of the dominant metallic component of 42CrMo4 steel, iron, were studied. Iron was detected in the oxidation states Fe (II) and Fe (III) (Figure 5) for all surfaces studied. Considering that the Fe(II)/Fe(III) ratio would be 1/2 for Fe_3_O_4_,the formation of stoichiometrically ideal Fe_3_O_4_ was only determined for LCM-H_3_PO_4_-6W. In contrast, for ground as well as LCM-NaNO_3_-6W and LCM-NaNO_3_-18W 42CrMo4 steel, deviations from the stoichiometrically ideal Fe_3_O_4_ were identified. For ground 42CrMo4 steel, the Fe(II)/Fe(III) ratio is almost 1/1, whereas for LCM-NaNO_3_-6W and LCM-NaNO_3_-18W 42CrMo4 steel surfaces it is close to 1/3. Therefore, the density of Fe(III) vacancies in Fe_3_O_4_ is assumed to be increased for ground surfaces. In contrast, the density of Fe(III) vacancies in Fe_3_O_4_ is decreased and increased for Fe(II) vacancies for LCM-NaNO3-6W and LCM-NaNO3-18W surfaces.

Additionally, metallic iron was detected on both ground and LCM-H_3_PO_4_-6W surfaces (Figure 5). The proportion of metallic iron is about 20% for ground 42CrMo4 steel and about 10% for LCM-H_3_PO_4_-6W. It is suspected that metallic iron from the base material has been detected by XPS. This suggests that the total thickness of the oxide layer on ground and LCM-H_3_PO_4_-6W is less than the measurement depth of the XPS, which is in the range of 10 nm. On LCM-NaNO_3_-6W and LCM-NaNO_3_-18W, no metallic iron was detected indicating an increased oxide layer thickness.

A qualitative analysis of the oxidation states of chromium, molybdenum and phosphorus was performed as well. Chromium is predominantly present as Cr(III) on all surfaces. Molybdenum was observed for the Mo(IV), Mo(V) and Mo(VI) states as well as in the metallic state on LCM-H_3_PO_4_-6W surfaces. For the other three surfaces, no additional information on the oxidation states of Mo is available due to the low contents of molybdenum. Furthermore, phosphorus is present as PO_4_^3−^ on LCM-H_3_PO_4_-6W surfaces.

**Figure 6:**
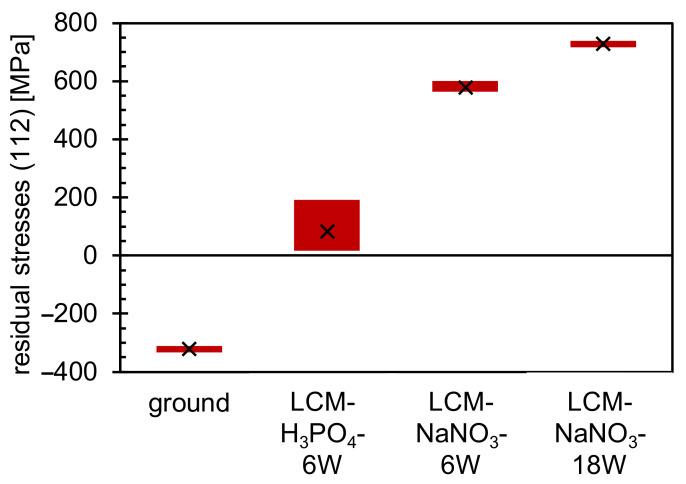
Residual stress measurements of ground and LCM 42CrMo4 steel.

Residual stress measurements were performed by XRD (Figure 6) to identify the influence of LCM on the stress development with varying process parameters. It was demonstrated that compressive residual stresses are present in the ground specimens, but tensile residual stresses are present in all LCM surfaces. The tensile residual stresses of the surfaces machined in NaNO_3_ solution are significantly higher than those of the surfaces machined in H_3_PO_4_. Furthermore, the tensile residual stresses are higher for LCM-NaNO_3_-18W in comparison to LCM-NaNO_3_-6W.

**Figure 7:**
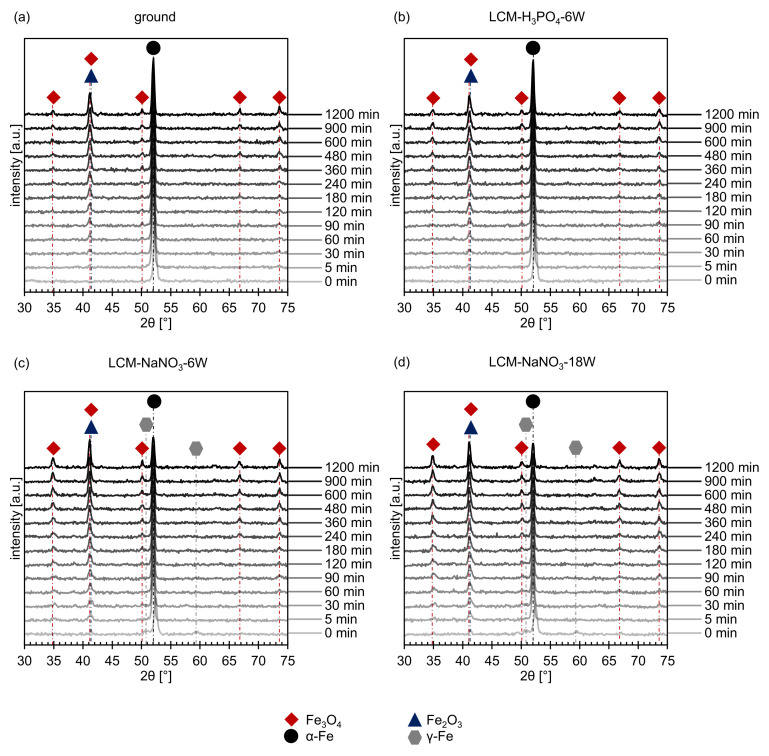
In situ XRD spectra of (**a**) ground, (**b**) LCM-H_3_PO_4_-6W, (**c**) LCM-NaNO_3_-6W and (**d**) LCM-NaNO_3_-18W 42CrMo4 steel at 500 °C up to 20 h.

In addition to the residual stress analysis, a phase analysis was performed on all four surfaces using XRD. On the ground and LCM-H_3_PO_4_-6W surfaces, only the (011) peak of α-Fe could be detected, as demonstrated in Figure 7a,b at 0 min. On the surfaces that were laser-chemically machined in NaNO_3_ solution, small amounts of iron oxide and γ-Fe were detected in addition to α-Fe (Figure 7c,d at 0 min). However, the identification of the oxides by XRD is challenging because the two main oxides, Fe_3_O_4_ and Fe_2_O_3_, exhibit overlapping peaks. In particular, the (113) peak of Fe_3_O_4_ at 41.0° and the (110) peak of Fe_2_O_3_ at 41.7° are difficult to separate. However, due to the fact that no other possible peaks of Fe_2_O_3_ were measured, it is assumed that an iron oxide with the structure Fe_3_O_4_ is the main component of the oxide at the surface of the two samples. This is consistent with the EDS and XPS measurements presented earlier (Figure 4 and Figure 5). Nevertheless, the presence of Fe_2_O_3_ cannot be completely excluded.

### 3.2. Oxide Formation at 500 °C

In situ XRD was used to study the influence of the different surfaces and rim zones on the formation of oxides at 500 °C for 20 h in more detail. The short-term experiments revealed on one hand the formation of Fe_3_O_4_ for all investigated rim zones (Figure 7). On the other hand, the overlapping of the (113) Fe_3_O_4_ peak and the (110) Fe_2_O_3_ peak might indicate the additional formation of Fe_2_O_3_. Since further Fe_2_O_3_ peaks are not present and may be suppressed by the background, it is assumed that mainly Fe_3_O_4_ is formed.

The intensity of the measured iron oxide peaks increases with increasing oxidation time for all investigated surface and rim zone conditions, while the intensity of the metallic α-Fe peak decreases. In addition, no γ-Fe was detected anymore for LCM-NaNO_3_-6W and LCM-NaNO_3_-18W 42CrMo4 steel surfaces already after 5 min of oxidation. This indicates either the transformation of the metastable γ-Fe phase or an increased oxide growth (Figure 7c,d).

**Figure 8:**
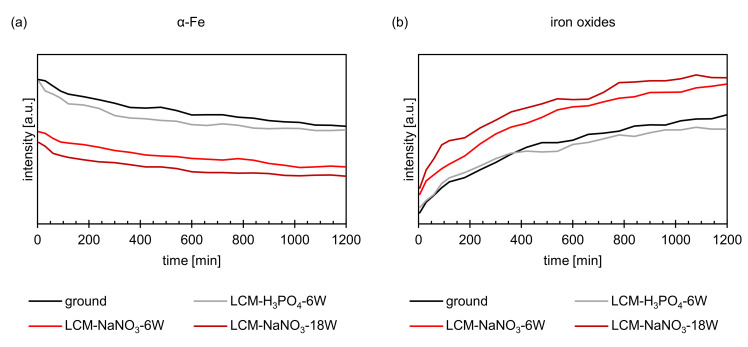
XRD intensities of (**a**) α-Fe and (**b**) iron oxides during oxidation at 500 °C up to 20 h.

Figure 8 shows the summation of all intensities of the iron oxide and metallic iron peaks as a function of the oxidation time obtained by in situ XRD. The total intensity of the metallic iron decreases with increasing oxidation time (Figure 8a), while the intensity of iron oxides (Figure 8b) increases and oxide growth occurs. Comparing the intensities of the formed oxides on ground and LCM-H_3_PO_4_-6W 42CrMo4 steel, it becomes visible that the oxidation follows a similar kinetic. In contrast, LCM-H_3_PO_4_ 42CrMo4 steel shows a significantly enhanced oxide growth compared to the ground surface. Furthermore, the oxidation kinetic of LCM-NaNO_3_ 42CrMo4 steel accelerates with increasing laser power.

**Figure 9:**
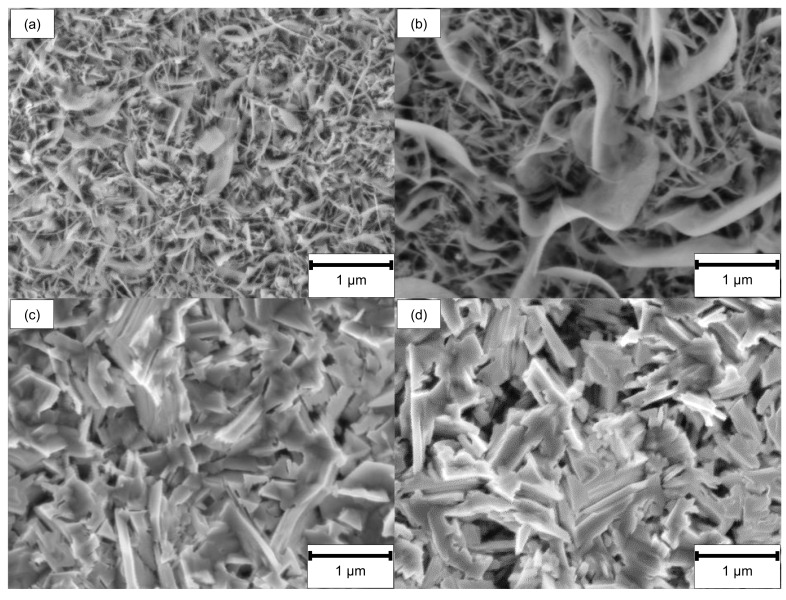
SEM top-view images after oxidation of (**a**) ground, (**b**) LCM-H_3_PO_4_-6W, (**c**) LCM-NaNO_3_-6W and (**d**) LCM-NaNO_3_-18W 42CrMo4 steel at 500 °C for 20 h.

SEM investigations revealed significant differences between ground and LCM-H_3_PO_4_-6W 42CrMo4 steel compared to LCM-NaNO_3_-6W and LCM-NaNO_3_-18W after oxidation at 500 °C for 20 h. SEM top-view images (Figure 9a,b) show the formation of whisker-like structure on the ground surfaces and the surfaces machined in H_3_PO_4_. In contrast, no whiskers but plate-like structures are present on both LCM-NaNO_3_ surfaces, as shown in Figure 9c,d.

**Figure 10:**
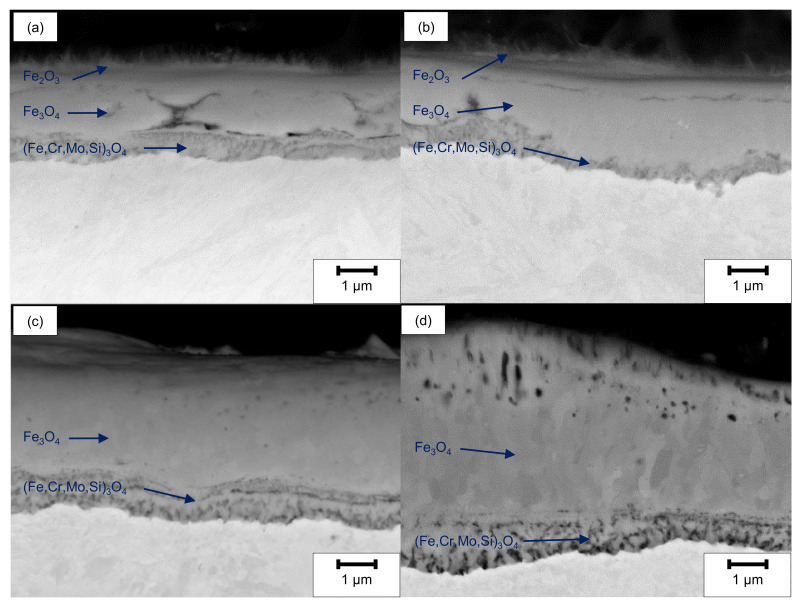
SEM cross-section images after oxidation of (**a**) ground, (**b**) LCM-H_3_PO_4_-6W, (**c**) LCM-NaNO_3_-6W and (**d**) LCM-NaNO_3_-18W 42CrMo4 steel at 500 °C for 20 h.

**Figure 11:**
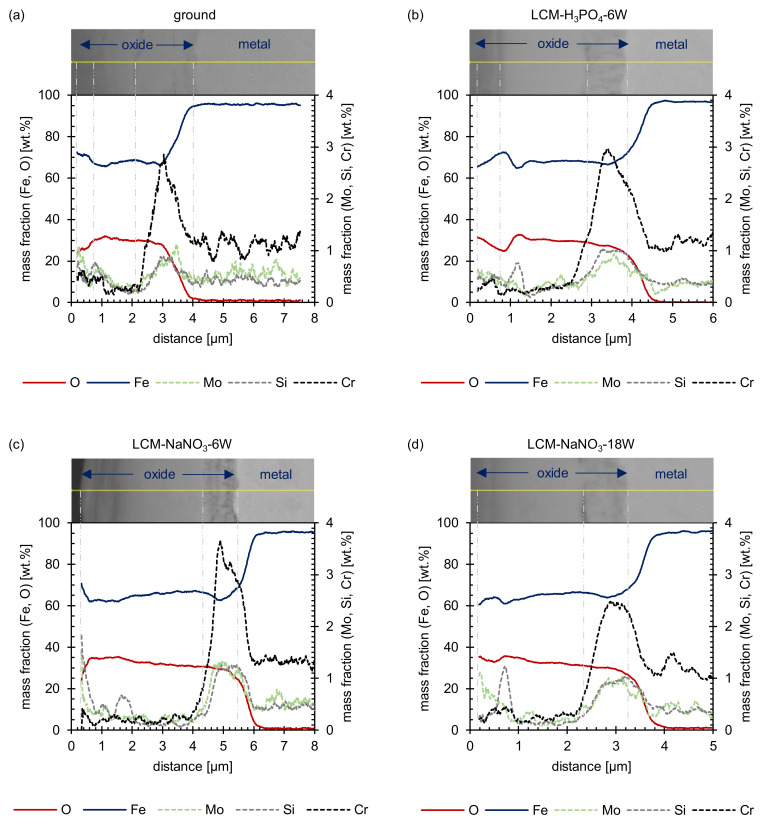
EDS line scans after oxidation of (**a**) ground, (**b**) LCM-H_3_PO_4_-6W, (**c**) LCM-NaNO_3_-6W and (**d**) LCM-NaNO_3_-18W 42CrMo4 steel at 500 °C for 20 h.

The SEM cross-sections (Figure 10) reveal the formation of several individual layers after oxidation at 500 °C for 20 h. Whereas for ground and LCM-H_3_PO_4_-6W surfaces three oxide layers were observed, only two oxide layers were formed for LCM-NaNO_3_-6W and LCM-NaNO_3_-18W. A porous inner layer of (Fe,Cr,Mo,Si)_3_O_4_ with an iron deficit and an enrichment of chromium, molybdenum and silicon was formed for all investigated rim zones. Generally, the second layer is also present for all investigated rim zone conditions. Mainly, a dense layer was observed with some porosity in the upper area. Silicon is partially enriched in these pores, as demonstrated in Figure 11, which is related to residues of the used polishing agent and the embedding material. Furthermore, a depletion of the alloying elements chromium and molybdenum was observed for the second layer, which is mainly composed of Fe_3_O_4_. In contrast to LCM-NaNO_3_-6W and LCM-NaNO_3_-18W, the ground and LCM-H_3_PO_4_-6W surfaces formed a third outer oxide layer (Figure 10a,b) after oxidation at 500 °C for 20 h. The composition and structure of the layer could not be detected by EDS and XRD. However, it is assumed on the basis of the generally accepted layer model of oxide formation on iron that this layer is composed of Fe_2_O_3_. The whisker-like morphology [21] may confirm this assumption.

**Figure 12:**
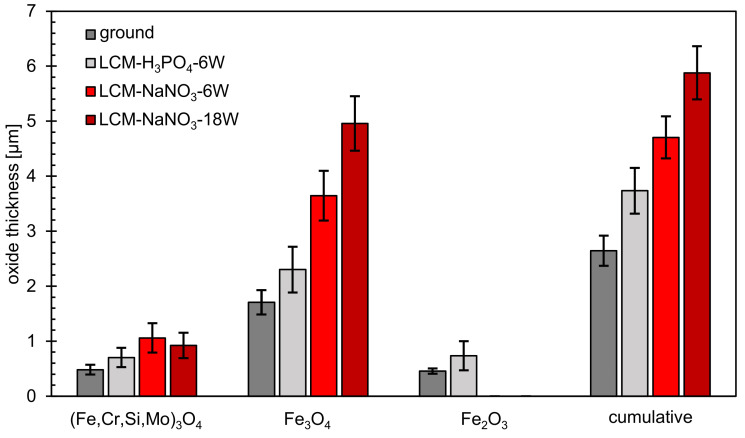
Thickness of oxide layers after oxidation at 500 °C for 20 h.

In addition to SEM and EDS analysis of the oxide films formed, the film thicknesses were also evaluated (Figure 12). This measurement showed that the total layer thickness increases from ground 42CrMo4 steel via LCM-H_3_PO_4_-6W and LCM-NaNO_3_-6W to LCM-NaNO_3_-18W surfaces. Thereby, the inner (Fe,Cr,Mo,Si)_3_O_4_ layer is of similar thickness for all surfaces studied. However, it was demonstrated that the second Fe_3_O_4_ layer of the LCM-NaNO_3_-6W to LCM-NaNO_3_-18W specimens is significantly thicker than the Fe_3_O_4_ layer on ground and LCM-H_3_PO_4_-6W surfaces. It is also noticeable that with increasing laser power, the thickness of the Fe_3_O_4_ layer increases for the LCM of 42CrMo4 steel in NaNO_3_ solution.

## 4. Discussion

### 4.1. LCM Rim Zone before Oxidation

The influence of machining parameters on rim zone properties, such as residual stresses and chemical composition, is an important aspect to understand the change of oxidation mechanisms by LCM rim zone modifications. A significant influence of the electrolyte on the surface integrity of 42CrMo4 steel after LCM was demonstrated by this study. The LCM-NaNO_3_-6W and LCM-NaNO_3_-18W surfaces exhibit a partially hill-shaped and irregularly covered surface layer of iron oxide with a maximum thickness of about 10 µm, while the LCM-H_3_PO_4_-6W surface is only covered by an oxide layer with a thickness of about 10 nm. Reasons for the varying oxide thickness are on one hand the differences in the heat input by the laser into the surface and on the other hand the change of the ablation mechanism due to the different chemical reactions due to the chosen electrolytes. Considering the influence of the heat impact, it was reported that changes in the surface integrity, such as roughness and chemical surface compositions, lead to a change in the laser absorption coefficient [34]. This might result in less heat input into the material during LCM in H_3_PO_4_ compared to NaNO_3_ solution for the same laser power. Furthermore, chemical reactions resulting in an increased formation of, e.g., hydrogen gas [35] will result in less heat being introduced into the material for H_3_PO_4_ compared to NaNO_3_ electrolyte at the same laser power. The hypothesis that less heat is introduced into the surface in NaNO_3_ electrolyte compared to H_3_PO_4_ at the same laser power is also strengthened by the fact that for LCM-NaNO_3_-6W a temperature-induced transformation of the microstructure from martensite to austenite was detected. Such a microstructural transformation was not observed for LCM-H_3_PO_4_-6W. In addition, the observed tensile residual stresses induced by the thermal impact during the LCM process are decreased for LCM-H_3_PO_4_-6W compared to LCM-NaNO_3_-6W and LCM-NaNO_3_-18W, indicating a lower heat input into the surface. Focusing on the composition of the formed oxide layers after LCM, an enrichment of chromium at the surface was observed by XPS. This is related to the thermodynamically higher oxidation tendency of chromium compared to iron at elevated temperatures [36].

Taking into account the influence of the chemical reactions on the observed differences of the rim zone properties, the choice of the different electrolytes, namely, H_3_PO_4_ and NaNO_3_, must be considered. The XPS investigations revealed the formation of PO_4_^3−^ and an enrichment of the oxide layer with molybdenum, chromium and silicon for LCM-H_3_PO_4_-6W 42CrMo4 steel. The formation of metal phosphates and the enrichment of the passive layer with molybdenum and chromium were already demonstrated for the exposure of 316L SS steel to H_3_PO_4_ by Prabakaran et al. [37] and confirms the assumption of the formation of a metal phosphate also for 42CrMo4 steel during LCM in H_3_PO_4_. XPS and REM-EDS measurements of the oxides on LCM-NaNO_3_-6W and LCM-NaNO_3_-18W 42CrMo4 steel also revealed an enrichment of chromium, molybdenum and silicon in the layer. Furthermore, the rim zone exhibits a very thin overstoichiometric Fe_3_O_4_ layer for ground compared to an almost stoichiometrically formed Fe_3_O_4_ layer on LCM-H_3_PO_4_-6W 42CrMo4 steel as well as a thick understoichiometric Fe_3_O_4_ layer for both LCM-NaNO_3_-6W and LCM-NaNO_3_-18W 42CrMo4 steel. The difference in the oxide thickness may also be attributed to the ability of H_3_PO_4_ to dissolve Fe_3_O_4_ and Fe_2_O_3_ compared to NaNO_3_. Summarizing, a strong influence of both the heat impact and chemical reactions depending on the selected electrolytes leads to the observed differences in the surface chemical composition, while only heat exposure affects the residual stress.

### 4.2. Oxidation Mechanism in Air at 500 °C

The microstructural results after oxidation at 500 °C for 20 h in air confirmed a strong influence of the rim zone properties obtained by LCM, such as residual stress and chemical composition, on the oxidation mechanism of 42CrMo4 steel.

In general, up to three layers were formed after the oxidation of ground and LCM 42CrMo4 steel at 500 °C for 20 h. More specifically, the growth of three oxide layers, composed of (Fe,Cr,Mo,Si)_3_O_4_, Fe_3_O_4_ and Fe_2_O_3_, were observed for ground and LCM-H_3_PO_4_-6W 42CrMo4 steel. In contrast, the formation of only two oxide layers, composed of (Fe,Cr,Mo,Si)_3_O_4_ and Fe_3_O_4_, was identified on LCM-NaNO_3_-6W and LCM-NaNO_3_-18W 42CrMo4 steel after oxidation.

The formation of the inner oxide (Fe,Cr,Mo,Si)_3_O_4_ is in good agreement with previous studies on Cr-Mo steels by other authors [18,20,21]. The inner oxide layer is riddled with many cracks and appears to have a scale-like structure. The Fe_3_O_4_ layer also contains cracks and pores, but to a smaller extent. These are predominantly located at the edges of the outer Fe_3_O_4_ layer. According to Trindade et al. [38], the differences in chemical composition, the number and distribution of cracks/pores and the structure between inner and outer oxide layer are due to different factors. First, the growth direction of the inner and outer oxide layers is different. The outer layer grows outwards and depends on the diffusion of iron ions from the material through the inner layer. The inner oxide layer, on the other hand, exhibits inward growth. Here, the diffusion of oxygen through the oxide layer towards the metal is the rate-determining step. The oxidation occurs first at the grain boundaries of the metal and then expands, which explains the scaly, cracked structure of the inner oxide layer. Second, the cracks are also due to stresses at the metal/oxide interface. Since the different oxide layers and the metal have different mechanical properties, the interfaces between metal and (Fe,Cr,Mo,Si)_3_O_4_ and between Fe_3_O_4_ and (Fe,Cr,Mo,Si)_3_O_4_ are particularly affected by cracks. [38]

In the area of the cracks and pores, contamination with elements such as silicon can also be detected partially in the upper area of the Fe_3_O_4_ layer. These contaminations are most likely impurities from the embedding material and the polishing agent.

In addition to the formation of (Fe,Cr,Mo,Si)_3_O_4_,Fe_3_O_4_, an additional outer Fe_2_O_3_ layer was observed on the ground and the LCM-H_3_PO_4_-6W surfaces after oxidation at 500 °C for 20 h. This layer is characterized by a whisker-like morphology. However, due to the small dimensions of the layer, neither the structure (by XRD) nor the chemical composition of this layer (by EDS) could be determined. However, based on the generally accepted layer formation model for the oxidation of iron and low alloyed steels in oxygen-containing atmospheres at about 500 °C, the formation of Fe_2_O_3_ is assumed [16,18]. In contrast, no formation of this additional Fe_2_O_3_ layer occurs on the surfaces processed in NaNO_3_. This is attributes to the fact that stoichiometrically, Fe_2_O_3_ contains less iron and more oxygen than Fe_3_O_4_. The transformation of Fe_3_O_4_ to Fe_2_O_3_ is therefore favored by either an iron deficit or an oxygen excess. The iron deficit depends on the diffusion rate of iron in the oxide. [39,40] For this reason, iron diffusion is faster through the oxide layer of LCM-NaNO_3_-6W and LCM-NaNO_3_-18W 42CrMo4 steel compared to LCM-H_3_PO_4_-6W and the ground surfaces. This prevents an iron deficit in Fe_3_O_4_ and therefore the formation of Fe_2_O_3_. In addition, this would also explain the increased thickness and growth rate of the Fe_3_O_4_ layer compared to LCM-H_3_PO_4_-6W and the ground surfaces, which is also significantly dependent on iron diffusion. The increased diffusion of iron in the oxide is attributed to an increased number of cation defects.

The increased thickness of the oxide layer on LCM-NaNO_3_-6W and LCM-NaNO_3_-18W in comparison to the ground and LCM-H_3_PO_4_-6W surfaces may also be due to the fact that a significant amount of a Fe_3_O_4_-like oxide was already present on these two surfaces prior to oxidation at 500 °C. According to Kuroda et al. [41], during the high-temperature oxidation of iron and steel, initial oxide nuclei, including Fe_3_O_4_, are formed in an incubation phase of a few minutes on the blank metal surface. Further oxide growth then subsequently starts from these initial nucleation sites. If Fe_3_O_4_ oxides are already present before oxidation, it is assumed by the authors that these Fe_3_O_4_-like oxides can act as a nucleation site for further layer formation during high-temperature oxidation. Consequently, in the case of LCM-NaNO_3_-6W and LCM-NaNO_3_-18W, this would lead to accelerated oxide layer growth. This is also indicated by the in situ XRD measurements. Already at the beginning of the oxidation, LCM-NaNO_3_-6W and LCM-NaNO_3_-18W 42CrMo4 steel revealed significantly higher intensities of Fe_3_O_4_ compared to ground and LCM-H_3_PO_4_-6W surfaces, which indicates a greater thickness of the oxide already in the initial phase of the oxidation.

Additionally, the increased residual tensile stresses in the LCM-NaNO_3_-6W and LCM-NaNO_3_-18W 42CrMo4 steel rim zone may contribute to increased film growth by promoting the diffusion of oxygen along the metal grain boundaries into the metal, thus promoting internal oxidation. However, the diffusion of oxygen along the grain boundaries of the metal mainly exhibit an influence on the growth of the inner (Fe,Cr,Mo,Si)_3_O_4_ oxide layer, since only this part of the layer grows from the outside to the inside [38]. For this reason, the differences in thickness of the outer Fe_3_O_4_ layer may not be attributed to the difference in residual tensile stresses. Increased defect density in the microstructure, which is caused by strong residual stresses, can additionally accelerate the diffusion of elements such as chromium from the base material to the oxide. However, Khanna et al. [42] found for the oxidation of 2¼Cr-1Mo steel in air that this only leads to a significant change in the oxidation properties (increased incorporation of chromium into the oxide layer) at temperatures of about 800 °C and higher. Thus, this effect is negligible for the present study.

Furthermore, an influence of the austenitic phase formed during LCM-NaNO_3_-6W and LCM-NaNO_3_-18W on the oxidation mechanism may occur. Since the austenitic phase was no longer detectable by in situ XRD at 500 °C after 5 min, the austenitic phase is assumed to mainly influence the oxidation initiation and not the growth. Possible influences of the locally observed austenitic microstructure on the oxidation are the changed chemical composition or modifications of the grain boundary density before and during oxidation [38]. This would, in turn, change the diffusion properties. However, a long-term influence of the austenitic phase on the oxidation mechanism is not likely and was not investigated in detail within the scope of this work.

The enrichment of chromium, molybdenum and silicon, which was observed before oxidation in the rim zone of LCM compared to ground 42CrMo4 steel, exhibits no significant influence on the oxidation mechanism. No enhanced incorporation of the alloying elements chromium and molybdenum into the (Fe,Cr,Mo,Si)_3_O_4_ oxide layer for LCM-NaNO_3_-6W, LCM-NaNO_3_-18W and LCM-H_3_PO_4_-6W surfaces was detected by EDS after oxidation. Moreover, such an enrichment of the (Fe,Cr,Mo,Si)_3_O_4_ oxide layer with chromium and molybdenum would lead to an improvement of the protective effect of the layer [15]. This leads to an overall lower oxide layer growth, which is in opposition to the results of this study. This also applies to the protective effect of small amounts of phosphorus, which has been described by Vannerberg et al. [43]. In LCM-H_3_PO_4_-6W, phosphates are present on the surface, which should improve the oxidation resistance of the material compared to the ground surfaces. However, this cannot be confirmed by the present measurements. In summary, it is suggested that the main influence on the differences in the oxidation mechanism is the variation in surface chemistry by LCM, which is achieved by the processing parameters and affects the process of oxidation initiation. Subsequently, this should lead to a different growth mechanism by obtaining different vacancy densities.

## 5. Conclusions

The rim zone properties generated by LCM have a significant effect on the oxidation behavior of 42CrMo4 steel below the wüstite temperature at 500 °C. LCM in NaNO_3_ solution at the process parameters chosen in this work (laser power 6 W and 18 W) results in a rim zone that is characterized by a cracked, porous layer of an Fe_3_O_4_-like oxide, in some cases several µm thick, as well as by strong tensile residual stresses. For the LCM process in H_3_PO_4_, on the other hand, only a few nm thick layers of iron oxide and phosphates were detected at the surface. Tensile residual stresses are also present, albeit weaker.

The following observations were made in regard to the oxidation mechanism:

- The thickness of the oxide layer is larger for LCM-NaNO_3_-6W and LCM-NaNO_3_-18W than for ground and LCM-H_3_PO_4_-6W surfaces. This is assumed to be predominantly attributed to the presence of Fe_3_O_4_-type oxides from the LCM process, which serve as oxidation nucleation sites at the beginning of the oxidation and thus accelerate the oxide layer growth.
- For all surfaces examined, an inner oxide layer of (Fe,Cr,Mo,Si)_3_O_4_ and an outer oxide layer of Fe_3_O_4_ could be detected. For the ground and LCM-H_3_PO_4_-6W surfaces, an additional outer Fe_2_O_3_ layer was identified. This layer is non-existent on LCM-NaNO_3_-6W and LCM-NaNO_3_-18W. This is attributed to the effect that iron diffusion is faster through the oxide layer for LCM-NaNO_3_-6W and LCM-NaNO_3_-18W than in LCM-H_3_PO_4_-6W 42CrMo4 steel and the ground surfaces. Since Fe_3_O_4_ preferentially converts to Fe_2_O_3_ in the presence of an iron deficit, rapid iron diffusion in the oxide can delay the formation of such iron deficiencies and thus the formation of Fe_2_O_3_.

In further work, the oxidation mechanisms will be analyzed in more detail. In particular, the influence of the individual rim zone properties (e.g., residual stresses, surface chemistry and roughness) on the oxidation initiation and growth will be investigated in isolation from one another. In addition, further investigations at temperatures above the wüstite (FeO) temperature are ongoing.

## Figures and Tables

**Table 1:** Chemical composition of 42CrMo4 steel adapted with permission from ref. [28]. Copyright 2020 Borchers et al., licensee MDPI, Basel, Switzerland.

Element	Cr	Mo	Mn	C	Si	Fe
**wt.%**	1.09	0.24	0.74	0.45	0.26	bal.

**Table 2:** LCM process parameters.

Process	Electrolyte	Electrolyte Volume [L]	Electrolyte Flow Rate [L/min]	Laser Power [W]
LCM-NaNO_3_-6W	2.5 M NaNO_3_	2	4	6
LCM-NaNO_3_-18W	2.5 M NaNO_3_	2	4	18
LCM-H_3_PO_4_-6W	5 M H_3_PO_4_	2	4	6

**Table 3:** Chemical composition of the uppermost rim zone prior to oxidation measured by XPS.

Element [wt.%]	Fe	O	Cr	Mo	C	P	N
ground [30]	52.0	31.5	1.0	<0.5	11.0	n.e.	n.e.
LCM-H_3_PO_4_-6W	34.5	42.0	1.5	3.5	12.5	6.0	n.e.
LCM-NaNO_3_-6W	33.5	40.0	6.0	<0.5	14.0	n.e.	<1.0
LCM-NaNO_3_-18W	35.5	42.5	3.0	<0.5	11.5	n.e.	<1.0

n.e.: not examined.

## Data Availability

The data presented in this study are available on request.
